# Seed fatty acid composition and physical dormancy in fire-prone ecosystems

**DOI:** 10.1093/aob/mcaf225

**Published:** 2025-09-18

**Authors:** Sarah J McInnes, Ryan Tangney, Mark K J Ooi

**Affiliations:** Centre for Ecosystem Science, School of Biological, Earth and Environmental Sciences, University of New South Wales, Sydney, New South Wales 2052, Australia; Centre for Ecosystem Science, School of Biological, Earth and Environmental Sciences, University of New South Wales, Sydney, New South Wales 2052, Australia; Department of Biodiversity, Conservation, and Attractions, Kings Park Science, Biodiversity, and Conservation Science, Kings Park, Western Australia 6005, Australia; Centre for Ecosystem Science, School of Biological, Earth and Environmental Sciences, University of New South Wales, Sydney, New South Wales 2052, Australia

**Keywords:** Seed biology, Fabaceae, fatty acid, gas chromatography–mass spectrometry, fire ecology, physical dormancy, fire-prone ecosystem, heat shock, seed chemistry, seed traits

## Abstract

**Background and Aims:**

The maintenance of seed banks and timing of germination are fundamental to ensuring population persistence. Physical dormancy (PY) in disturbance-prone environments contributes to these processes via an impermeable seed coat. Dormancy is broken often by heating, which in fire-prone regions is determined by species-specific threshold temperatures. However, the mechanisms by which seeds persist or control dormancy-breaking thresholds in such environments are unclear. We determined whether unsaturated and saturated fatty acids (FAs; within triacylglycerols), common lipids linked to heat-stress resilience, might contribute to seed coat dormancy and overall seed persistence, and whether fire selects for different FA compositions and drives PY function in fire-prone regions.

**Methods:**

We characterized seed FA compositions of 26 Fabaceae species from fire-prone and fire-free ecosystems through gas chromatography–mass spectrometry. We compared FA saturation, total relative FA content and the highest melting point FA of each species across seed tissues (seed coat vs internal tissues) and habitat type (fire-prone vs fire-free) and, for fire-prone species, tested for a relationship with species-specific dormancy-breaking thresholds.

**Key Results:**

No relationship between FA composition and species-specific dormancy-breaking thresholds was found. Seeds of fire-free species had more saturated FAs than fire-prone species, particularly for internal tissues. FA saturation was higher in seed coats than in internal tissues across both habitat types. Relative FA content was similar in internal tissues across habitat type but differed for seed coats, with fire-prone species having marginally more FAs.

**Conclusions:**

While no correlation existed between FA composition and dormancy-breaking thresholds in fire-prone species, the consistent differences between seed tissue types we found highlight a similar role for FAs in seed coats across habitats, probably linked to maintaining impermeability. Some evidence supports fire selecting for greater total FA content in seed coats, but further work is needed to test its relationship with temperature thresholds.

## INTRODUCTION

Fire is an ecological driver, directly influencing ecosystem structure and function over millennia (Keeley et al., 2011; He and Lamont, 2018). Plant species within fire-prone ecosystems have evolved under distinct fire regimes (Keeley *et al*., 2011), allowing them to persist and cue key life-history stages, such as germination, with the extreme temperatures associated with fire. However, climate change is altering the fire regimes plant species are adapted to (Miller *et al*., 2019; IPCC, 2022; Tangney *et al*., 2022; Le Breton *et al*., 2023), increasing the risk of population decreases for species unable to cope with the new conditions (Nolan *et al*., 2021; Le Breton *et al*., 2022). Thus, there is an imperative to research the fundamental mechanisms behind the life-history traits of plant species that support persistence in fire-prone ecosystems, to better understand how species might function or be limited, under various fire regimes (Keith, 2012; Jiménez-Alfaro *et al*., 2016).

The maintenance and release of seed dormancy are important traits for plant species (Nolan *et al*., 2021). In fire-prone ecosystems, seed dormancy contributes to the maintenance of seed banks, allowing population persistence by ensuring seeds are available to germinate into the post-fire environment to replace plants that are killed by fire (Whelan, 1995; Miller and Murphy, 2017). Species that produce seeds with physical dormancy (PY) have seed coats that are impermeable to water. The loss of this impermeability (via dislodging the water–gap complex or cracks forming in the seed coat; see Morrison *et al*., 1998; Gama-Arachchige *et al*., 2013) causes dormancy to break, which then enables water to enter and promote germination (Baskin *et al*., 2000). For PY seeds in regions that do not regularly experience fire (arid and coastal dune regions, hereafter ‘fire-free’), large fluctuations in daily temperature typically rupture the seed coat and render it permeable (Baskin and Baskin, 2014). In fire-prone environments, fire-related temperatures between 60 and 150 °C can also release PY as high heat shock breaks the seed coat, rendering it permeable (Keeley, 1987; Herranz *et al*., 1998; Merritt *et al*., 2007; Moreira and Pausas, 2012; Ooi *et al*., 2014; Tangney *et al*., 2025). These fire-related heat shocks are considered more effective at breaking PY in fire-prone regions than seasonal temperature fluctuations (Moreira and Pausas, 2012; Pausas and Lamont, 2022; but see also Jaganathan, 2015). In some environments, PY break is reported to occur in a two-step process: preconditioning, as seeds are made sensitive to dormancy break (i.e. sensitivity cycling, see Jayasuriya *et al*., 2008, 2009), then dormancy break itself as seeds become water-permeable (Taylor, 1981). However, the role of sensitivity cycling in fire-prone regions is currently understudied and there is conflicting evidence as to its importance in these systems (Lamont *et al*., 2022, 2024; Luna *et al*., 2023).

The duration of PY break also differs across fire-free and fire-prone ecosystems, occurring across months and within minutes respectively (Jaganathan and Harrison, 2024). These temporal differences in PY break are due to the negative relationship between temperature and exposure duration, as higher temperatures require less time to break PY (Pausas and Lamont, 2022; Jaganathan and Harrison, 2024). However, when exposure times are constant, there is still considerable inter-specific variation in dormancy-breaking temperatures (Auld and O’Connell, 1991; Tangney *et al*., 2025) and the exact mechanism that drives this variation in dormancy-breaking thresholds between species is unknown. Both methods of PY break in each habitat type relate to gap-detection (and thus reduced competition) and are timed for when germination conditions are optimal (Baskin and Baskin, 2014) – in fire-prone regions, this is the post-fire environment (Pausas *et al*., 2022). PY is considered a bet-hedging strategy, as seed germination is staggered over time, thus accounting for unpredictable environmental conditions in any given year. However, it has been argued that PY in fire-prone ecosystems operates using a best-bet strategy, as a large portion of the soil seed bank germinates due to the predictability of suitable germination conditions in the post-fire landscape (see Pausas and Lamont, 2022; Pausas *et al*., 2022).

**Fig. 1.**
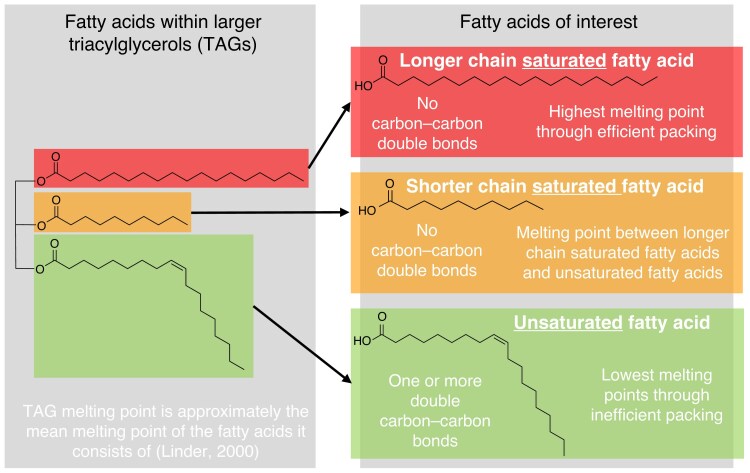
Saturated and unsaturated fatty acids (FAs) from larger triacylglycerols (TAGs) captured in this study and relative melting points. Melting points are determined by FA structure where increasing carbon chain length and a reduction in carbon–carbon double bonds increase individual FA melting point. The TAG melting point is the mean of the FA melting points (Linder, 2000).

Fatty acids (FAs) and their composition within different tissues of PY seeds may contribute to the functioning of seed dormancy in fire-prone ecosystems, as high melting point FAs are reported to assist in maintaining cell functioning at high temperatures (Russell, 2003). FAs are a common lipid present in plant tissues and accumulate in lipid bodies within the seed as the macromolecule triacylglycerol (TAG; Huang, 1985), making up approximately 90 % of all lipids present (Krist *et al*., 2005). They are also present as minor components in other lipid macromolecules and polymers such as waxes and cutins in the seed coat, where they are suspended within the cellulose matrix (Werker, 1981). FA composition also varies across cell type (Harwood, 1980) and the melting points of FAs are similar to that of the whole TAG (Linder, 2000) – henceforth, throughout this paper, discussion of FAs refers to those found within TAGs. Generally, as the carbon chain length of the FA increases, the melting point of the FA also increases. Moreover, saturated (i.e. no carbon–carbon double bonds) FAs have significantly higher melting points than unsaturated (i.e. one or more carbon–carbon double bonds) FAs (Fig. 1) (Blackman and Gahan, 2014).

The seed coat and embryo have distinct FA compositions across seeds from all dormancy types (Harwood, 1980) and perform different roles in each of these structures (Baskin *et al*., 2000). In the seed coat, FAs can be found in larger macromolecules that are important for maintaining seed coat impermeability, such as cutin, waxes and TAGs (Shao *et al*., 2007; Sano *et al*., 2016; Simpson *et al*., 2016; Chai *et al*., 2021). It has been hypothesized that FA melting points in the seed coat correlate positively with the temperature that breaks dormancy in PY seeds (Zeng *et al*., 2005; Hudson *et al*., 2015; McInnes *et al*., 2023). This hypothesis has been proposed because, in fire-prone regions, dormancy-breaking temperatures can be distinct between individual species and this variation is a driver of community assemblage (Hudson *et al*., 2015). Seed coats of species from fire-prone habitats also have distinct FA compositions compared to species from regions without regular fire, as high melting point FAs (exceeding 70 °C) are more prevalent in the seed coats of fire-prone species (McInnes *et al*., 2023). This suggests that FAs may play a role in PY seed coats that is specific to fire, as air and soil temperatures exceeding 70 °C are rare in the absence of fire (Ooi *et al*., 2009, 2012). If FAs do contribute to dormancy-breaking temperatures, we hypothesize that species with the most FA saturation and longest saturated FA carbon chains in the seed coat, and thus the highest FA melting points, also have the highest dormancy-breaking temperatures.

FAs in the seeds themselves (i.e. the embryo, endosperm and cotyledons) are primarily catabolized to provide energy during seedling germination in oily seeds (not only those with PY; Harwood, 1980). The proportion of saturated FAs within a seed is related to climate (Linder, 2000), where saturated FAs are associated with warmer, lower latitudes, and unsaturated FAs are more prominent in seeds from colder, higher latitude climates. However, the role that FA composition may play within seeds for species from fire-prone environments, beyond that related to fuelling germination, is less well understood. It has been suggested that increased FA saturation can help individual persistence at high temperatures by maintaining cell membrane fluidity through stronger intermolecular bonding (Russell, 2003; Upchurch, 2008; Visscher *et al*., 2021; Zhang *et al*., 2021). During fire, the increased stability conferred by higher FA saturation may be important to preserve critical material contained by the internal tissues – such as the embryo – and thus allow for subsequent germination following fire. If FAs within the seed (i.e. embryo, cotyledons and endosperm) contribute to PY functioning in fire-prone ecosystems, it would be expected that a higher proportion of saturated FAs were present in PY seeds from fire-prone ecosystems due to exposure to higher temperatures compared to seeds from habitats that rarely experience fire.

Here we investigated FA compositions (including both saturated and unsaturated FAs) in PY seeds of Fabaceae, comparing species from either fire-prone ecosystems or ecosystems that do not regularly experience fire (‘fire-free’). We have expanded on our previous work (McInnes *et al*., 2023) by investigating FA composition in both the seed coat and seed embryo, endosperm and cotyledons (hereafter referred to as ‘internal tissue’) to broadly characterize FA profiles in the different seed tissue types and attempt to uncover novel relationships between FA composition and PY in fire-prone and fire-free environments. Our key aims were to:

1. Explore whether patterns of FA composition differ across fire-prone and fire-free habitats for the two seed tissue types and discuss what the identified patterns tell us about the potential functions of seed FA composition.
2. Determine if seed coat FA melting points positively correlated with species-specific dormancy-breaking thresholds in fire-prone species.

**Fig. 2.**
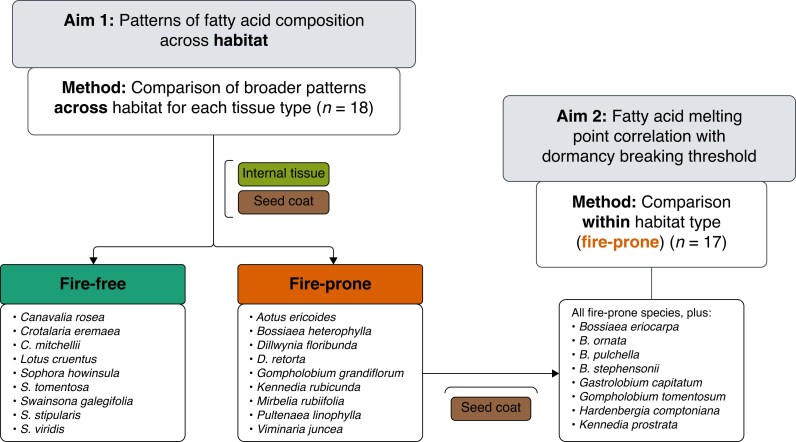
Outline of the aims and methods of this study, created in Lucid (lucid.co).

We set out to address our two aims (Fig. 2), ultimately aimed at understanding the role of FAs in the functioning of dormancy in PY seeds.

## MATERIALS AND METHODS

### Species selection

We used 26 species, all from the subfamily Faboideae in the family Fabaceae, which are common in temperate fire-prone, arid and coastal dune ecosystems in Australia. Species were selected from habitats that either experienced fire regularly (fire-prone) or extremely rarely (fire-free). Fire-prone species used in this study occur in habitats that have recommended minimum fire intervals of between 7 and 30 years by the NSW Rural Fire Service, whereas the fire-free species are primarily from regions where burning is not recommended (coastal dune and arid shrublands: chenopod sub-formation) or occasionally from regions which have a minimum interval recommendation of 10 years but no maximum threshold (arid shrublands: acacia sub-formation; NSW Rural Fire Service, 2024). Seeds were either commercially purchased or collected directly from the parent plant across 2009–2018 and dry seeds stored under ambient laboratory conditions (Supplementary Data Table S1; Fig. S1).

To assess patterns in FA composition in the seed coat and internal tissues across habitat type (Aim 1, Fig. 2), we compared seed FAs of nine fire-prone and nine fire-free species from New South Wales, Australia. To determine whether FA melting points correlated with species dormancy-breaking thresholds (Aim 2, Fig. 2), we used solely fire-prone species from across New South Wales (*n* = 10) and Western Australia (*n* = 7), using only seed coat FA data. As dormancy-break in PY seeds is caused by the dislodgement of water–gap complexes in the seed coat (Gama-Arachchige *et al*., 2013), internal tissues were not used for the analysis between dormancy-breaking thresholds in fire-prone species and FA composition. Existing seed coat FA data were taken from McInnes *et al*. (2023; seed coat data for 23 species). Remaining seed FA data (18 species internal tissue data, three species seed coat data) were collected using the same method as McInnes *et al*. (2023), which is summarized below.

### Fatty acid extraction and identification

To extract and identify FAs present within seed coats and internal seed tissues, analysis was conducted on dormant seeds. As PY is lost over time (Van Assche and Vandelook, 2006; Orscheg and Enright, 2011), intact seed dormancy was ensured by soaking seeds in water overnight (16–24 h; Pascualides and Planchuelo, 2007; Varela and Albornoz, 2013): those that floated (i.e. were probably inviable; Cochrane *et al*., 1999) or had imbibed water (i.e. were non-dormant; Liyanage and Ooi, 2018) were removed. PY seeds (i.e. seeds that had an intact, water-impermeable seed coat preventing germination; Baskin and Baskin, 2014) were separated into internal tissues and seed coat using a scalpel and forceps, ground down (∼ 50 mg), and stirred in hexane (3 mL) for 1 h to extract the lipids. Sodium methoxide was added (in methanol; 0.15 mL) and stirred for 10 min to isolate the FAs from the TAG and esterify them into fatty acid methyl esters (FAMEs), which are more readily analysed by gas chromatography–mass spectrometry (GC-MS). Solutions were then filtered through cotton wool to remove remnant seed. FAs were identified and quantified relatively using GC-MS at the Bioanalytical Mass Spectrometry Facility within the Mark Wainwright Analytical Centre of the University of New South Wales.

Samples were run on a Thermo Scientific DSQ II GC-MS with an Agilent polar HP88 column (30 m × 0.25 mm × 0.25 µm) and MS in electron impact positive ionization mode. The GC ran from 40 °C (30 s hold) to 115 °C at 7.5 °C min^–1^, then from 115 °C to 240 °C at 3.5 °C min , with a 5-min hold at 240 °C. Helium was the carrier gas at a constant flow rate of 1 mL min^–1^. The inlet and MS detector temperatures were 250 and 230 °C respectively, and the injection split ratio 10:1. The MS transfer line temperature was 245 °C, and scans were from 34 to 450 *m*/*z* at 3.5 scans s^–1^. To identify FAMEs, mass spectra of each FAME were compared against reference spectra in the Wiley and NIST Mass Spectral libraries. Matches above 80 % that also included characteristic FAME peaks at 74 and 87 *m*/*z* were deemed accurate identifications. Matches below this threshold were additionally checked against a FAME standard (FAME Mix C8-C24, Sigma-Aldrich) to confirm the identification based on retention time. The total ion current traces of each FAME peak were integrated to calculate relative quantities using OriginPro v.10.0.0.154 (OriginPro, 2023). Samples were normalized by sample weight.

### Comparison of FA composition between the seed coat and internal tissues of fire-prone and fire-free species

To visualize FA composition variation in our study, we used factor analysis of mixed data (FAMD). We included the continuous (proportion saturated FAs, relative total FA content) and categorical dependent FA variables (longest saturated FA, dominant FA), and the categorical predictor variable habitat type (fire-free, fire-prone), repeating the analysis for each seed tissue type (seed coat, internal tissue). Three fire-free species were excluded from the seed coat analysis (*Sophora tomentosa*, *Swainsona galegifolia*, *Crotalaria mitchelli*), as total relative FA content in these species was not comparable to data collected in McInnes *et al*. (2023) due to differences in GC-MS responsiveness. FAMD analysis was conducted using the *FactoMineR* v.2.8 (Lê *et al*., 2008) and *factoextra* v.1.0.7 (Kassambara and Mundt, 2020) packages, and results were plotted using these and the package *ggplot2* v.3.4.2 (Wickham, 2016). Figures were collated using *ggpubr* v.0.6.0 (Kassambara, 2023).

To assess patterns in different seed tissue FA profiles across habitat, we examined two variables that were the most likely to be important in contributing to PY: longest saturated FA and proportion saturated FAs. Differences in FA compositions between habitat type (fire-prone vs fire-free) and seed tissue type (seed coat vs internal tissue) were examined for 18 species (nine fire-prone, nine fire-free) via multiple linear models. All analyses were performed in the statistical platform R v.4.3.1 (R Core Team, 2024). Between habitats, data were first split according to seed tissue type (internal tissue vs seed coat). Saturated FA proportion was then Tukey transformed (Mangiafico, 2024) for both the internal tissue and seed coat and compared against habitat type. Within habitat type, the FA composition of the internal tissue and seed coat was compared. Saturated FA proportion was log-transformed in fire-free habitats, and square root-transformed in fire-prone environments. The total relative FA content was also compared across habitat type for the internal tissue (*n* = 9) using a linear model and log-transformation. Seed mass was also compared against total relative FA content, proportion saturated FAs and species dormancy-breaking temperatures through multiple linear models and log-transformations (Supplementary Data Tables S2–S4; Figs S2 and S3). Transformations were done to ensure a normal distribution, which was checked through a Shapiro–Wilk test of normality via the inbuilt *stats* v.4.3.1 package.

### Calculating dormancy-breaking threshold temperature

To assess the correlation between FA melting points and dormancy-breaking thresholds among fire-prone PY seeds, we first estimated the dormancy release temperature for 17 species. For each species, the dormancy-breaking threshold temperature was defined as the temperature when 50 % of viable, dormant seeds germinated (Tangney *et al*., 2025, but similar metrics used in Ooi *et al*., 2014). Raw data were taken from Liyanage and Ooi (2018), Tangney *et al*. (2025), and Auld and O’Connell (1991), where seed lots were exposed to temperatures ranging from 40 to 140 °C for either 5 or 10 min, and germination was recorded.

Species-specific dormancy release temperatures were calculated using methods outlined in Tangney *et al*. (2025) . In brief, the final germination proportion across each heat-shock treatment was used to create a thermal performance curve (TPC) for each species using the package *rTPC* v.1.0-2 (Padfield *et al*., 2024). Multiple non-linear models from the *rTPC* package were fitted to each curve and the most suitable model was selected based on visual inspection and the lowest Akaike information criterion ranking. The selected model for each species had the residuals bootstrapped (100 iterations) using the *minpack.lm* v.1.2-3 and *car* v.3.1-2 packages (Elzhov *et al*., 2016; Fox and Weisberg, 2019) to generate a 95 % confidence interval (CI) surrounding the dormancy-release temperature. Non-viable proportions were subtracted from each treatment (as reported in Auld and O’Connell, 1991 and Liyanage and Ooi, 2018, whereas viability was checked pre-treatment in Tangney *et al*., 2025) and non-dormant proportions accounted for during analysis (see Tangney *et al*., 2025), thus ensuring only dormant and viable seeds were used when calculating the dormancy threshold. Data were taken from treatments that were either 5 or 10 min, as thresholds were assumed to be similar at these times (Auld and O’Connell, 1991).

### Dormancy release temperature analysis

Once the dormancy release temperature had been defined and calculated, we tested whether either of the FA variables (saturated FA proportion and longest saturated FA, which both relate to FA melting point) correlated with the dormancy-breaking temperature of the 17 fire-prone species. Generalized linear models (GLMs) were used to compare each FA variable against species’ dormancy-breaking thresholds, and a negative binomial distribution was used to account for over-dispersion using the *MASS* v.7.3-58.2 package (Venables and Ripley, 2002) in R v.4.3.1 (R Core Team, 2024). The analysis was repeated for each variable and results were plotted using the *ggplot2* v.3.4.2 (Wickham, 2016) and *sjPlot* v.2.8.14 packages (Lüdecke, 2021), then collated with *ggpubr* v.0.6.0 (Kassambara, 2023). Saturated FA proportion was Tukey transformed (Mangiafico, 2024) to normalize the distribution, and the longest saturated FA in each species was treated as a factor as only two distinct FAs were present.

## RESULTS

### Grouping of FA composition variance by habitat across seed tissue type

**Fig. 3.**
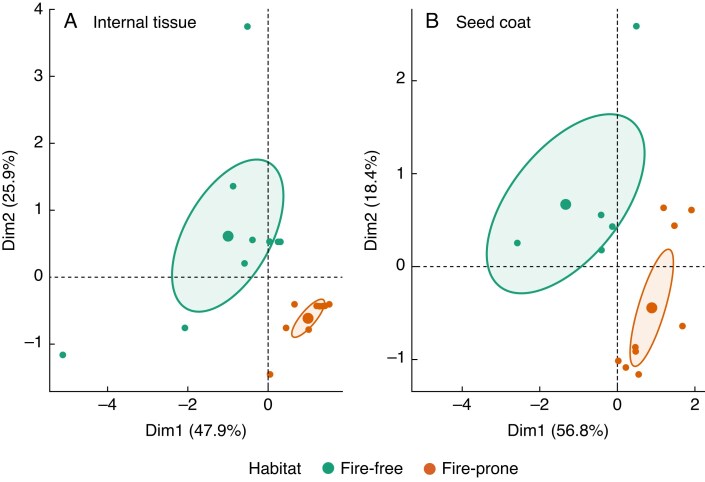
Factor analysis of mixed data (FAMD) showing the grouping of fatty acid composition variance across habitat and seed tissue type.

Characterizing the patterns in FA composition across habitat type in the seed coat and internal tissue resulted in clear separation across the two axes, where FA saturation appeared to be a key driver of variation across habitats. In the internal tissue, the first two principal components (PCs) explained 74 % of the variation (Fig. 3). The strongest contributions to PC1 (47.9 %) were FA saturation (38 %) and the dominant FA (27 %), and PC2 (25.9 %) had strong contributions from relative FA total (57 %) and habitat (23 %). In the seed coat, the first two PCs explained 75 % of the variation. PC1 (56.8 %) had contributions mainly from proportion saturated FA (38 %), dominant FA (27 %) and the longest saturated FA (18 %), whereas PC2 (18.4 %) was tied strongly with FA total (57 %) and habitat (25 %). This indicated that the drivers of variation in FA composition were similar in each tissue type where mainly FA saturation appeared to differentiate fire-prone and fire-free species along the PC1 axis – in particular, that fire-free species had a higher proportion of saturated FAs than fire-prone species within each tissue type. Relative FA total also contributed to habitat differentiation (57 % of PC2), although with lesser impact than FA saturation due to the lower variance explained by PC2 (18.4 %) compared to PC1 (56.8 %).

### Trends in FA composition across seed tissue type and habitat

**Fig. 4.**
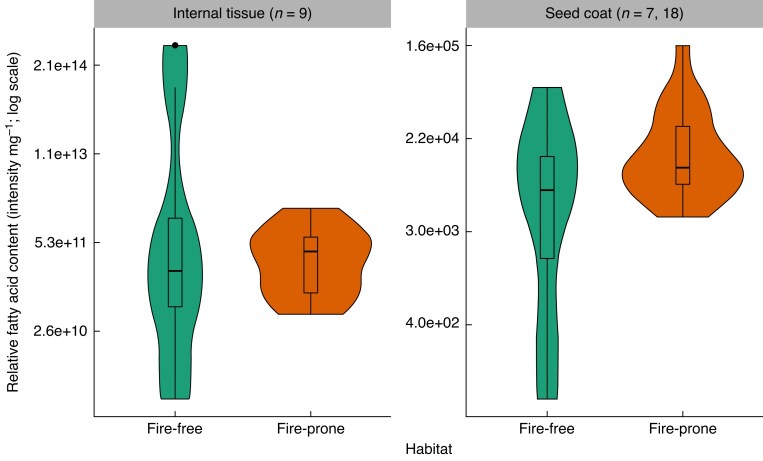
Relative total fatty acid (FA) for the internal tissues (left panel) and the seed coat (right panel; modified under a CC BY 4.0 license from McInnes *et al*., 2023), each separated by habitat type (fire-prone or fire-free). Data are presented on a log scale (see Supplementary Data Table S2), and *n* = 7 for fire-free and *n* = 18 for fire-prone species in the seed coat (right panel). Total FA did not differ significantly for internal tissues (left: F = 0.081, df = 1, P = 0.780) nor seed coats between habitats (right: χ2 = 3.811, df = 1, P = 0.051).

**Fig. 5.**
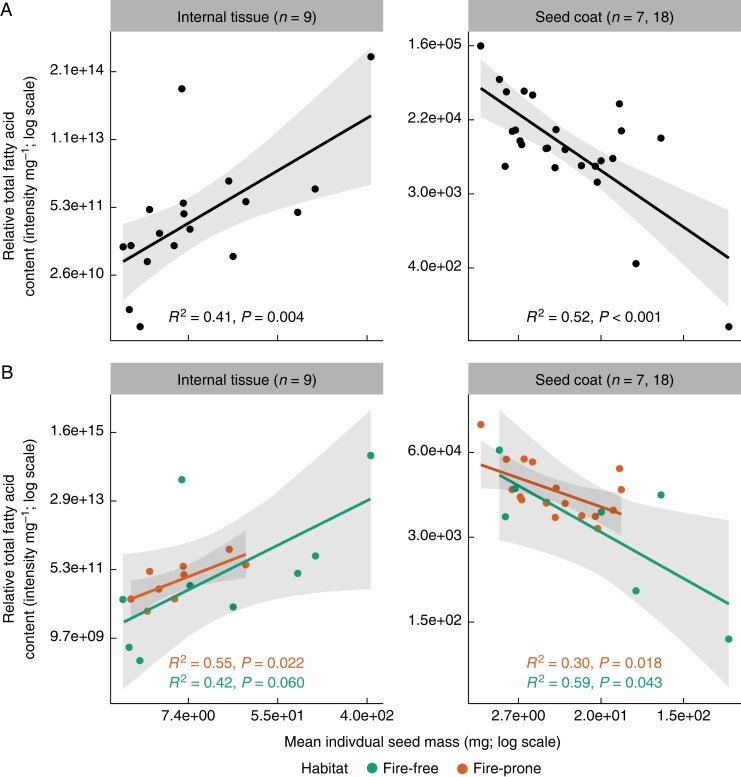
Relationship between mean individual seed mass and the relative total of fatty acids (FAs) across the internal tissue and seed coat with 95 % confidence intervals. Data are presented on a log scale (see Supplementary Data Table S2). (A) Mean individual seed mass positively correlated with the relative total internal tissue FAs (F = 11.2, df = 1, P = 0.004) and negatively correlated with total seed coat FAs (F = 28.9, df = 1, P < 0.001) across both fire-free and fire-prone habitats. (B) The relationship between seed mass and relative FA total was stronger in fire-prone than fire-free species (internal tissue fire-prone: F = 8.60, df = 1, P = 0.022; internal tissue fire-free: F = 5.04, df = 1, P = 0.060; seed coat fire-prone: F = 7.01, df = 1, P = 0.018; seed coat fire-free: F = 7.31, df = 1, P = 0.043).

The internal tissue had more FAs than the seed coat, but the total relative FA content in the internal tissue was similar across both habitat types (Fig. 4: *F* = 0.081, df = 1, *P* = 0.780). Relative total FA was positively correlated with seed mass in the internal tissue (Fig. 5A: *F* = 11.2, df = 1, *P* = 0.004), but had a negative relationship with seed mass in the seed coat (Fig. 5A: *F* = 28.9, df = 1, *P* < 0.001). These relationships were still present when split by species habitat (Fig. 5B).

**Fig. 6.**
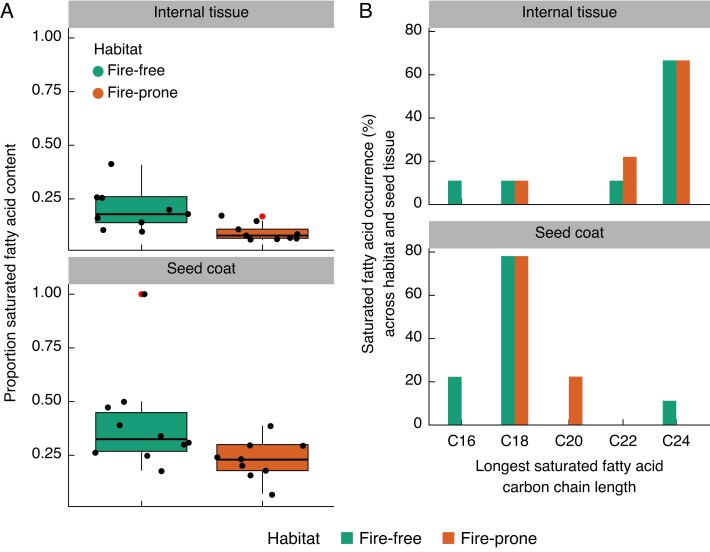
Comparisons between the fatty acid (FA) composition of the seed internal tissue and seed coat across fire-prone and fire-free habitats. (A) Boxplots of the saturated FA proportion in fire-prone and fire-free species, split by tissue type. Raw data are jittered and outliers are shown in red. Interquartile ranges (IQRs) for the internal tissue were 0.14 (fire-free) and 0.07 (fire-prone), and IQRs for the seed coat were 0.27 (fire-free) and 0.19 (fire-prone). Outliers were defined as values more than 1.5 times the IQR from either end of the box (see ‘geom_boxplot’ in the *gpplot2* package for further details; Wickham, 2016). (B) The occurrence (%) of individual saturated FAs within the internal tissue and seed coat, separated by habitat. Percentage occurrence was calculated based on whether a specific saturated FA occurred in a species (species were grouped by habitat and the corresponding FA compositions separated by tissue type, for a total of four groups) and the total occurrence within each group divided by the total number of species in that group (n = 9), and converted into a percentage. This was repeated for each distinct saturated FA. Seed internal tissues consisted of less saturated FAs (A: fire-prone: F = 17.1, df = 1, P < 0.001; fire-free: F = 9.17, df = 1, P = 0.008) and had longer fatty acids (B) than the seed coat.

**Fig. 7.**
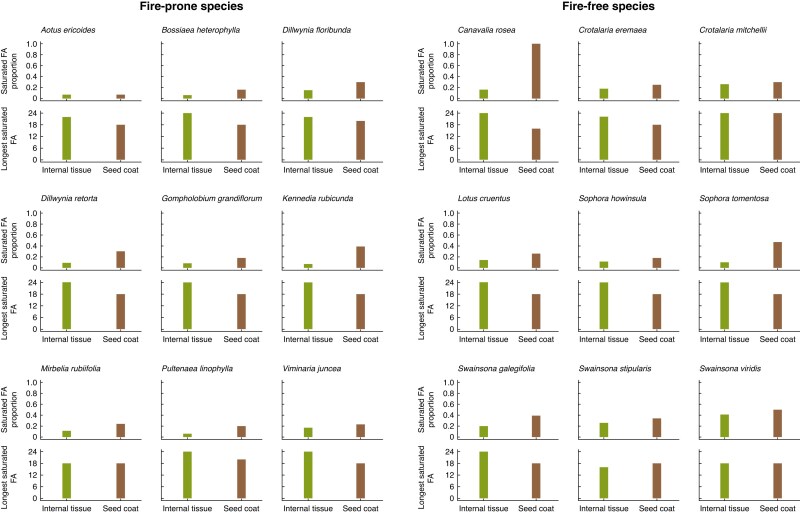
Individual species’ fatty acid (FA) profiles, separated by habitat. The proportion of saturated FAs and the longest saturated FA is shown for each species, for both the seed coat and internal tissue.

Supporting the FAMD results, where FA saturation was a key contributor to variation in FA composition across habitats, fire-free species had significantly more saturated FAs in both the seed coat and internal tissue than fire-prone species (Fig. 6A; internal tissue: *F* = 14.2, df = 1, *P* = 0.002; seed coat: *F* = 5.51, df = 1, *P* = 0.032), and a larger range of saturated FA chain lengths was found in fire-free species (Fig. 6B). Within the same habitat, seed coats were found to have significantly more saturated FAs (Fig. 6A; fire-prone: *F* = 17.1, df = 1, *P* < 0.001; fire-free: *F* = 9.17, df = 1, *P* = 0.008) compared to internal tissues from the same habitat. This increase in FA saturation from the internal tissue to the seed coat ranged from 4 to 84 %, with the only exception being the fire-prone species *Aotus ericoides*, where the internal tissue had 0.04 % more saturated FAs than the seed coat (7.47 and 7.43 % respectively; Fig. 7). However, low melting point unsaturated FAs were dominant across both tissue types, consisting of >50 % of all FAs in every species except the seed coats of the fire-free *Canavalia rosea* (0 % unsaturated FAs) and *Swainsona viridis* (49.7 % unsaturated FAs). The three most unsaturated FA compositions were all from the internal tissues of fire-prone species (Fig. 7: *Bossiaea heterophylla*, *Pultenaea linophylla* and *Kennedia rubicunda*).

The longest saturated FAs were consistently found in the internal tissue (Fig. 6B). The majority of species (17 out of 18) had maximum FA chain lengths in their internal tissues equal to or longer than the seed coat (with the exception of one species, *Swainsona stipularis*; Fig. 7). However, in the seed coat, no clear trend in maximum FA chain length across fire-prone and fire-free species was apparent, contrasting with previous work which showed longer FA chains in the seed coat (McInnes *et al*., 2023). Seed coats from fire-free species had both the longest (C24:0, *Crotalaria mitchellii*) and shortest (C16:0, *Canavalia rosea*) maximum FA chain lengths, whereas fire-prone species had maximum chain lengths confined to C18:0 and C20:0, with C20:0 only occurring in fire-prone species. Moreover, C18:0 was found to be the dominant maximum chain length in the seed coats of both fire-free and fire-prone species.

### FA melting point versus dormancy-breaking temperature thresholds in fire-prone species

**Fig. 8.**
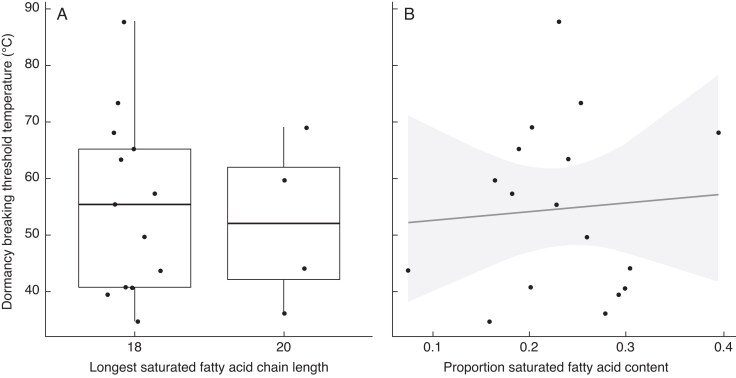
(A) The longest saturated fatty acid in each species, compared against the species-specific dormancy-breaking threshold temperature (χ^2^ = 0.142, df = 1, *P* = 0.707. (B) Proportion of saturated fatty acids in PY seed coats compared against species-specific dormancy-breaking threshold temperatures with 95 % confidence intervals. Proportions closer to 1 are indicative of more saturated fatty acids, whereas results closer to zero are indicative of more unsaturated fatty acids (χ^2^ = 0.094, df = 1, *P* = 0.759).

To compare seed coat FAs against dormancy-breaking thresholds for seeds in fire-prone regions, species-specific dormancy-breaking temperatures were calculated (Table 1). There were no significant differences found between dormancy-breaking thresholds and any of the FA dependent variables. No relationship existed between dormancy-breaking thresholds and the longest saturated FA, and thus highest FA melting point (Fig. 8A; χ^2^ = 0.142, df = 1, *P* = 0.707), and the median dormancy release temperatures were similar (52 and 55 °C) regardless of the longest saturated FA present. Furthermore, no statistically significant relationship was apparent between the proportion of saturated FAs in each species seed coat and dormancy-breaking threshold (Fig. 8B; χ^2^ = 0.094, df = 1, *P* = 0.759).

**Table 1.** Dormancy-breaking threshold temperature of species in fire-prone habitats, defined as the temperature where 50 % of dormant, viable seeds germinate (Tangney *et al*., 2025).

Species	Dormancy breaking threshold (°C; 95 % CI)
*Aotus ericoides*	43.8 (43.7, 44.0)
*Bossiaea eriocarpa*	55.4 (52.8, 58.3)
*Bossiaea heterophylla*	34.8 (29.7, 35.8)
*Bossiaea ornata*	40.9 (32.0, 46.3)
*Bossiaea pulchella*	65.2 (60.7, 69.4)
*Bossiaea stephensonii*	49.7 (49.3, 50.1)
*Dillwynia floribunda*	44.2 (42.7, 44.3)
*Dillwynia retorta*	40.7 (32.4, 48.1)
*Gastrolobium capitatum*	39.5 (29.6, 43.9)
*Gompholobium grandiflorum*	57.3 (52.4, 62.4)
*Gompholobium tomentosum*	59.7 (51.7, 68.2)
*Hardenbergia comptoniana*	36.2 (26.2, 41.6)
*Kennedia prostrata*	73.4 (70.5, 77.5)
*Kennedia rubicunda*	68.2 (67.1, 69.1)
*Mirbelia rubiifolia*	63.4 (63.0, 64.3)
*Pultenaea linophylla*	69.0 (65.1, 73.9)
*Viminaria juncea*	87.7 (86.1, 89.2)

Thresholds are reported alongside the 95 % confidence interval (CI).

## DISCUSSION

We found relationships between habitat of origin and FA composition, although these were not consistent across all dependent variables. While internal tissues were found to have similar total relative FA content when compared across habitat type, seed coats of species from fire-prone ecosystems have previously been shown to have more FAs than fire-free species (McInnes *et al*., 2023). Despite this, we found no significant relationship between seed coat FA composition and dormancy-breaking temperature thresholds for our fire-prone study species. When looking at the proportional distribution of FAs, fire-free species tended to have a greater proportion of saturated FAs than fire-prone species, particularly for internal tissues, suggesting that climatic variables rather than fire are driving germination-related FA composition as FA composition aligned with the germination temperatures of each habitat type (Linder, 2000). When comparing between seed tissue types, seed coats were found to have more saturated FAs and consisted of shorter saturated FAs than the internal tissues across both habitats.

### Grouping of variance across seed tissue type and habitat

The variance in FA composition in the seed coat and internal tissues for the study species partitioned according to habitat (fire-free and fire-prone) and was largely driven by FA saturation (i.e. the ratio of unsaturated and saturated FAs). The seed coat and internal tissues are known to have different FA compositions given their distinct roles (Harwood, 1980; O'Dowd and Gill, 1986), and there is evidence showing that seed coat FA composition can be distinct between fire-free and fire-prone regions (McInnes *et al*., 2023). Fire-free species showed more variation across PC1 in both tissue types (47.9 and 56.8 % of variance) than fire-prone species, which was strongly correlated with FA saturation. The fire-free species used in this study spanned three clades within the Faboideae (Genistoid, Robinoid, Inverted repeat-lacking clade/IRLC), which occur either in coastal or arid ecosystems, whereas fire-prone species spanned two clades (Millettoid and Mirbelioid) and only occur within dry sclerophyll forests. As whole-seed FA composition can be affected by both genetics and the environment (Zhang *et al*., 2015), it is likely that this variation is due to phylogenetic and/or environmental influences rather than fire-prone species inherently being less variable. The grouping of data according to both habitat and seed tissue type indicates that the observed variations in FA composition are probably linked to the general physiological functioning of the different seed tissues.

### FAs in the internal tissue and germination energy

The internal tissues of fire-free species had more saturated FAs than fire-prone species, a finding that does not support our hypothesis that fire-prone internal tissues would have more saturated FAs due to exposure to high fire-related temperatures. This suggests that event-driven impacts (i.e. high temperature heat shock from fire) or higher post-fire soil temperatures (e.g. Santana *et al*., 2010) do not drive internal seed tissue FA composition. An alternative hypothesis suggests that temperature is the key driver of FA variation in seed internal tissues, based on the relationship between FA saturation and latitude (Linder, 2000). Increased FA saturation is found in species from warmer areas because saturated FAs contribute energy towards germination more efficiently per carbon, but under lower germination temperatures are utilized more slowly than unsaturated FAs. For species with distributions across a range of latitudes, intra-specific variation in FA composition permits seeds to become better suited to local germination temperatures (Linder, 2000; Branham *et al*., 2016; Sanyal *et al*., 2018). The fire-free species in our study were primarily from arid ecosystems, where mean annual temperatures reach approximately 29 °C (Bureau of Meteorology, 2024). Conversely, the fire-prone species were from temperate dry sclerophyll forest where ambient temperatures are lower, with approximate mean annual temperatures of 22 °C (Bureau of Meteorology, 2024). This difference in mean annual temperature of approximately 7 °C could therefore potentially underlie the greater proportion of saturated FAs in the internal tissue of fire-free (18 %) compared to fire-prone (8 %) species.

Unsaturated FAs dominated the internal tissues of fire-prone species (92 % of all FAs). Additionally, the overall saturated FA proportion was lower in the internal tissues of fire-prone species when compared to the overall average recorded for Fabaceae species from temperate ecosystems (8 % vs 14 %; Linder, 2000). High proportions of unsaturated FAs in seeds are not uncommon and have been reported to range from 65 to 85 % in Fabaceae species (Franco *et al*., 2023). Unsaturated FAs provide less overall energy than saturated FAs but are catabolized more rapidly and allow for quicker growth in cooler environments, a potentially beneficial trade-off at higher latitudes (Linder, 2000; Branham *et al*., 2016). As all the fire-prone species used here experience lower germination temperatures than the fire-free species (Mackenzie *et al*., 2016; Ryan *et al*., 2023), the high degree of FA unsaturation is potentially due to the lower energy input required to catabolize unsaturated FAs in cooler environments (Zhang *et al*., 2015), providing the potential benefits of rapid establishment post-fire (Quintana *et al*., 2004; Verdú and Traveset, 2005). However, unsaturated FAs are at higher risk of degradation than saturated FAs through lipid peroxidation (Long *et al*., 2015; Diantina *et al*., 2022). Australian temperate dry sclerophyll forests have fire intervals of between 10 and 50 years (DPIE, 2021), requiring seeds to persist in the soil seed bank for extensive periods of time (e.g. an *Acacia suaveolens* seedbank was modelled to persist for 60 years; Auld, 1987). Native seeds have previously been found to consist of large quantities of unsaturated FAs (>50 %), but no relationship between FA composition, seed viability and seed ageing was present (Merritt, 2001). Thus, how seeds from fire-prone species balance unusually high proportions of unsaturated FAs in the internal tissue with long-term longevity in the soil seed bank remains unclear.

The positive correlation between seed mass and internal tissue relative FA content across both habitats also supports internal tissue FAs being predominantly associated with germination energy. FAs contained within the seed are a known energy source for germination (Harwood, 1980) and larger seeds were found to have more internal tissue FAs on a per milligram basis than smaller seeds. Larger seeds emerge from deeper within the soil, but deeper emergence depths require more energy input from seed storage reserves to successfully emerge from the soil (Fenner and Thompson, 2005). Thus, it follows that the larger seeds in this study had larger energy reserves compared to smaller seeds.

### Potential roles of seed coat FAs

The relative total FA content in internal tissue was similar across habitat type whereas previous results have found that the seed coats of fire-prone species contain marginally more FAs than fire-free seed coats (McInnes *et al*., 2023). This suggests the potential for a specialized role of seed coat FAs in different habitats and that fire may potentially drive selection for higher melting points, and therefore more saturated FAs, in the seed coats of fire-prone species. However, despite this, FA composition did not correlate with dormancy release requirements in PY seeds of our study species from fire-prone regions. Neither saturated FA chain length nor FA saturation correlated with dormancy-breaking temperature – and thus the cause of the variation in these thresholds remains unclear. It is possible that clearer trends were missed due to a small sample size (*n* = 17), and further work, perhaps including finer resolution dormancy-breaking thresholds across a larger number of species, would allow further testing.

When looking at the proportion of FA saturation, the seed coats of our study species had more saturated FAs and had shorter chain FAs than the internal tissue in both fire-free and fire-prone species, suggesting some similarities in the role that seed coats play independent of habitat type. The seed coat is not generally considered a major source of germination energy storage (Rajjou *et al*, 2012), and the negative relationship between seed mass and relative total FA content in the seed coat supports this as larger seeds require more, not less, energy to emerge from the soil (Fenner and Thompson, 2005). Rather, FAs in the seed coat tend to be found in larger macromolecules associated with seed impermeability (Sano *et al*., 2016), and thus the FA composition within the seed coat aligns with a different role than internal seed FAs.

Maintenance of a water-impermeable seed coat is critical in PY seeds as dormancy loss is an irreversible life-stage for PY seeds, as seeds rapidly hydrate and initiate germination if conditions are suitable or perish trying. PY is broken through the opening of the water–gap complex, which becomes dislodged after thermal stress (Gama-Arachchige *et al*., 2013) or physical abrasion. It has been hypothesized that a larger presence of unsaturated FAs, which are hydrophobic and liquid under ambient temperatures, may assist in maintaining PY through contributing to the protective, water-impermeable layer of the seed coat (McInnes *et al*., 2023). The hydrophobicity and mix of unsaturated and saturated FA melting points could also contribute to seed coat maintenance through similar mechanisms observed in *Banksia*, where waxes melt periodically to seal fissures and act as a self-sealing mechanism (Huss *et al*., 2018).

Contributions to water impermeability can exist through other pathways. Wholly saturated FAs (within a TAG) have been shown to exist within the hydrophobic cuticle of bayberry fruits (*Myrica pensylvanica*; Simpson *et al*., 2016). The synthesis pathway for these TAGs was more closely related to pathways for cutin, rather than typical TAG, synthesis. As such, the role of these TAGs was more similar to that of cutin, which can impose impermeability in crop legumes such as soybean (Shao *et al*., 2007) and is important in maintaining the hydrophobicity of the cuticle (Simpson *et al*., 2016). Given the high proportion of saturated FAs (mean 32 ± 5 %) and the general presence of FAs within the seed coat, similar pathways may exist for plant species that produce PY seeds.

Alternatively, increased FA saturation may contribute to general seed coat stability and dormancy maintenance. Higher FA saturation has been linked with increased survival at high temperatures in thermophilic bacteria (Russell, 2003), as saturated FAs pack together more efficiently than unsaturated FAs and thus have stronger inter-molecular bonding. Proportions of saturated FAs in the seed coats of fire-free and fire-prone species were 34 and 23 % respectively, which was higher than the internal tissue across both habitats and the overall mean of 14 % recorded for Fabaceae seeds in temperate regions (Linder, 2000). This higher FA saturation in the seed coat compared to the internal tissue may contribute to the overall maintenance of dormancy, as increased FA saturation may correspond to an overall strengthening of the seed coat through stronger inter-molecular bonding (Russell, 2003). This may also explain why FA saturation was higher in the seed coat of fire-free compared to fire-prone species. Mean annual temperature for fire-free species was 29 °C compared to 22 °C in the fire-prone species (Bureau of Meteorology, 2024), and thus increasing FA saturation in warmer regions may maintain dormancy during unfavourably hot germination conditions (fire-free species) or the inter-fire period (fire-prone species; Zomer *et al*., 2022). Ultimately, whether FAs in the seed coat contribute to PY maintenance through increased seed coat stability, maintaining water-impermeability, or something else entirely requires understanding how the FAs are structured and interact with other molecules within the seed coat cellular matrix.

### Dynamic changes in FA composition

Evidence for saturated FAs contributing to seed survival at high temperatures is conflicting (Kee and Nobel, 1985; Visscher *et al*., 2021). However, FA compositions are dynamic and change in response to maternal conditions (Graham *et al*., 1981; Feder and Hofmann, 1999). Under elevated temperatures, plants can alter the FA saturation within cell lipid membranes to maintain fluidity and thus cell functioning (Feder and Hofmann, 1999). Increased FA saturation maintains this fluidity under high temperatures, due to having stronger intermolecular bonds than unsaturated FAs, and has been observed in soybeans grown under experimental conditions (Wolf *et al*., 1982). More recently, FA saturation was shown to increase in tomato seeds exposed to 60 °C for 2 h (Ibrahim and El-Muqadam, 2019). A number of studies have also reported dynamic changes in FA saturation in response to temperature (Larkindale and Huang, 2004; Hou *et al*., 2006; Niu and Xiang, 2018), and others have reported no changes or conflicting results (Canvin, 1965; Kee and Nobel, 1985; Zheng *et al*, 2011). Given that plant FA composition can be dynamic in response to heat stress (Upchurch, 2008), it is possible that PY seeds alter their FA composition to cope with heat stress during and/or after fire. As this study only captured baseline seed FA compositions before exposure to fire-related temperatures, future work should sample FA composition before, during and after heat stress to account for the potential of a dynamic FA profile.

### Limitations and future directions

This study was limited by sample size when comparing across habitats (*n* = 18) and to species-specific dormancy-breaking thresholds (*n* = 17), thus further work with a larger number of species tested needs to be done to confirm the trends observed here. Moreover, the dormancy-breaking thresholds calculated for the fire-prone species used in our study were based on a different seed lot than those used in this study, albeit many had a similar provenance to those that were used in calculating dormancy-breaking thresholds (Sydney basin region; see Supplementary Data Table S1 and Auld and O’Connell, 1991; Liyanage and Ooi, 2018). The results should therefore be interpreted with caution, given that thermal thresholds can vary with seed age and population (Liyanage and Ooi, 2017; Overton *et al*., 2024). Nevertheless, this study is one of the first and largest to explore seed FA composition in the context of fire, and current results clearly demonstrate that FA composition is shaped by a species’ environment.

Further research should seek to test the FA composition of species that occur in both fire-prone and fire-free ecosystems, which would permit for a stronger test of fire as a driver of FA composition in PY seeds, alongside how phylogenetic relatedness (particularly the clustering of fire-prone and fire-free taxa) may influence observed FA patterns. Moreover, exploring the FA composition of specific cell types, relationships with other seed traits (e.g. seed coat thickness), whether FA composition relates to the temperature fluctuations required to break PY in fire-free species, alongside where and how FAs are structured within the seed and change in response to thermal stress and PY break, would allow for a deeper understanding of how FAs interact with PY.

## CONCLUSION

Ultimately, no correlation was found between FA composition and seed dormancy-breaking thresholds for fire-prone species, indicating that seed coat FA composition does not play a role in determining whether the temperature thresholds for PY break are reached during fire. However, FA composition differed within the internal tissue and seed coat across both fire-prone and fire-free ecosystems as seed coats had more saturated FAs and shorter FAs than the internal tissue. The consistency of these patterns across habitats suggests that FA composition within each tissue type is shaped by its environment, potentially to assist in the functioning of PY in that specific environment. Specifically, the FA composition of the internal tissues may be related to germination, as higher FA saturation was present in the internal tissues of fire-free species, which have higher germination temperatures than fire-prone species. Within the seed coat, FAs may contribute to maintaining water impermeability as no relationship with dormancy-breaking temperatures was present. If the mechanisms behind PY functioning during fire are understood, better predictions of population dynamics under climate-induced shifting fire regimes can be made.

## Supplementary Material

mcaf225_Supplementary_Data

## SUPPLEMENTARY DATA

Supplementary data are available at *Annals of Botany* online. Table S1. Seed provenance and sample size for fatty acid extraction. Table S2. Fatty acid total content of each species, split by tissue type. Table S3. Seed mass data. Table S4. Relative abundance of each fatty acid within each species, split by seed tissue type. Figure S1. Non-significant relationship between seed saturated fatty acid proportion and seed age. Figure S2. Seed mass against the proportion of saturated fatty acids within a species, split by tissue type and coloured by habitat. Figure S3. Seed mass against species-specific dormancy-breaking threshold temperatures.
