# Supplementary material for: Seed fatty acid composition and physical dormancy in fire-prone ecosystems
Source: Ann Bot. 2025 Sep 18;137(1):209–22. doi: 10.1093/aob/mcaf225 (PMC12784075; doi:10.1093/aob/mcaf225)
Supplement: mcaf225_Supplementary_Data [file mcaf225_supplementary_data.zip › supplementary_figures.pdf]

Supplementary Figures 1-3

Figure S1: Proportion saturated fatty acid content against seed age.

Figure S2: Seed mass against proportion saturated fatty acid content.

Figure S3: Seed mass against species-specific dormancy-breaking threshold temperatures.

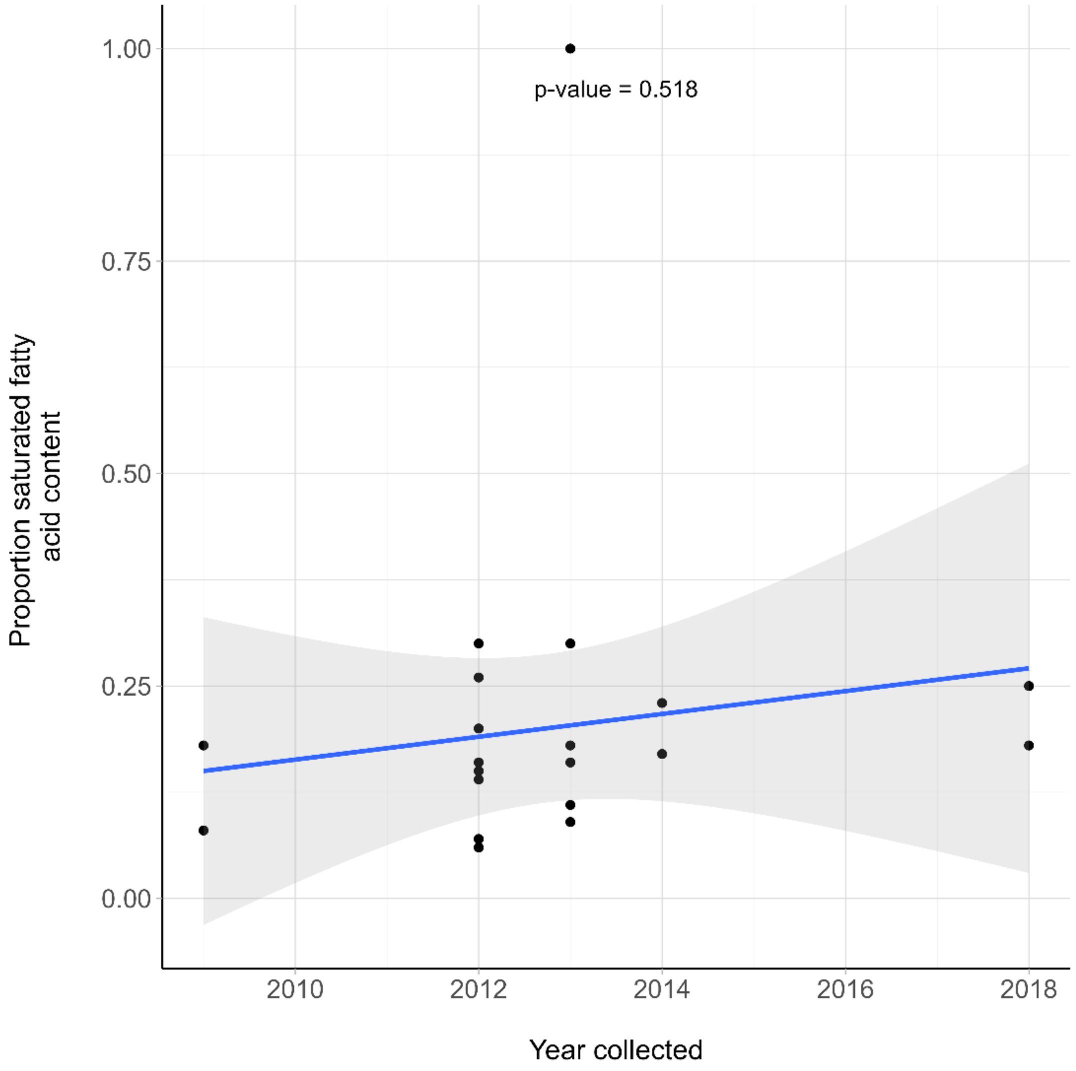

Supplementary Figure 1: Non-significant relationship between the proportion of saturated fatty acids against the year seeds were collected. Seed age data were not available for seeds purchased commercially. Statistical significance was calculated via a linear model ($F = 0.433$, $df = 1$, $p = 0.518$).

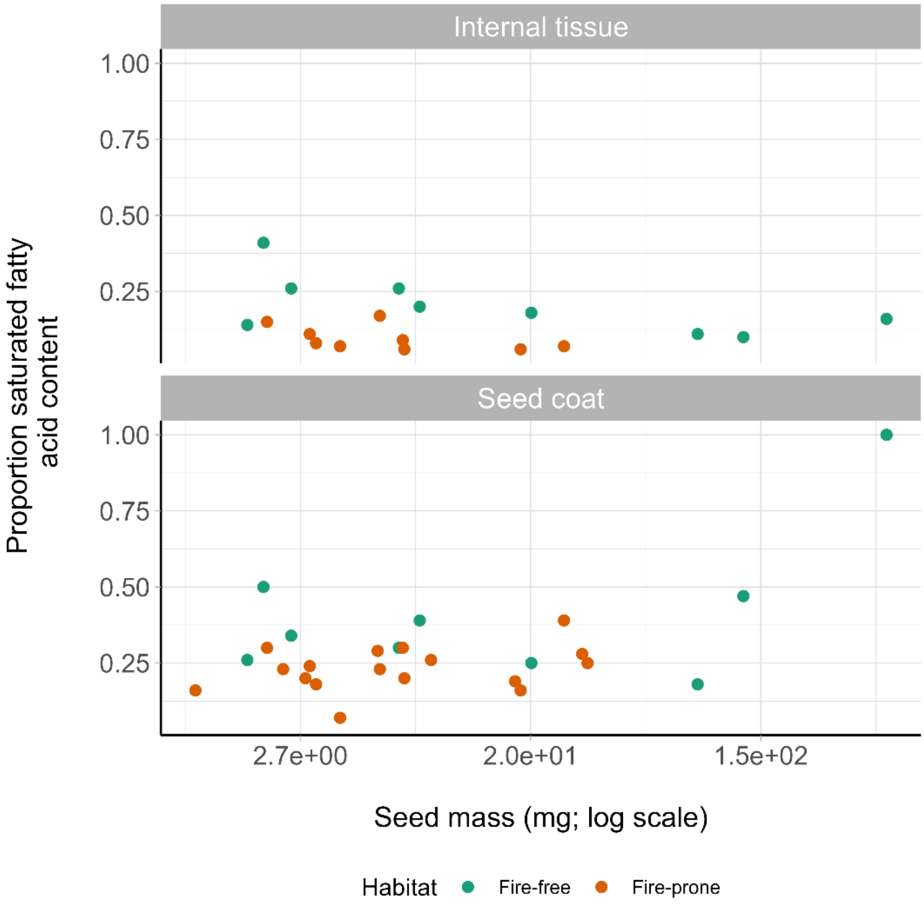

Supplementary Figure 2: Relationship between species-specific seed mass and the proportion of saturated fatty acids across the internal tissue and seed coat. Seed mass data are available in Table S3.

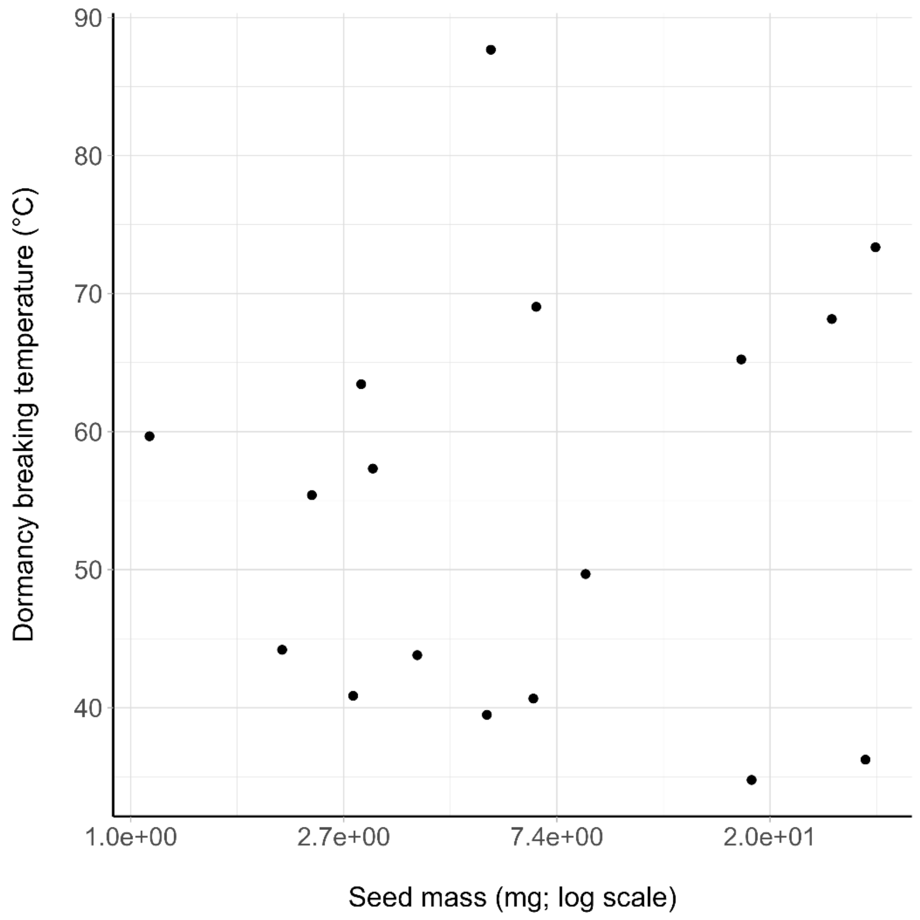

Supplementary Figure 3: Relationship between species-specific dormancy-breaking thresholds and seed mass for species in fire-prone ecosystems. Seed mass data are available in Table S3.
